# Supplementary material for: Shared Treg dysregulation underlies psoriasis vulgaris and atopic dermatitis through the PHF19-PRC2 epigenetic axis
Source: Front Immunol. 2026 Aug 20;17:1932788. doi: 10.3389/fimmu.2026.1932788 (PMC13538097; doi:10.3389/fimmu.2026.1932788)
Supplement: Supplementary file 3 [file DataSheet3.zip › Additional files/Additional file 8/66.FAM200B.leaveoneout.pdf]

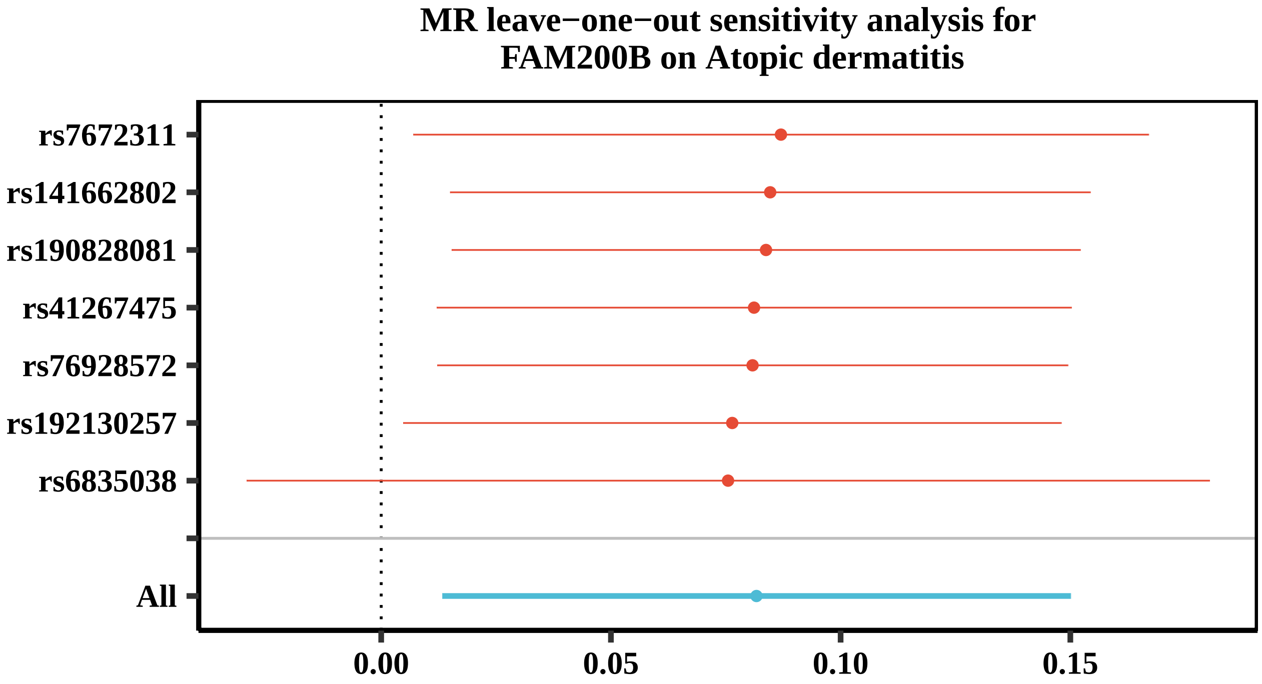
MR leave−one−out sensitivity analysis for
FAM200B on Atopic dermatitis
rs7672311
rs141662802
rs190828081
rs41267475
rs76928572
rs192130257
rs6835038
All
0.00
0.05
0.10
0.15
